# Programa Mais Médicos: caracterização da qualidade da atenção básica considerando o tipo de provimento médico

**DOI:** 10.1590/0102-311XPT115623

**Published:** 2024-11-22

**Authors:** Mozart Julio Tabosa Sales, Paulo Savio Angeiras de Goes, Aline Priscila Rego de Carvalho, Caio Cesar Arruda da Silva, José Roberto da Silva, Carlos Nobre e Silva, Fernando Antonio Menezes da Silva, Suely Arruda Vidal

**Affiliations:** 1 Instituto de Medicina Integral Prof. Fernando Figueira, Recife, Brasil.; 2 Faculdade Pernambucana de Saúde, Recife, Brasil.; 3 Faculdade de Odontologia de Pernambuco, Universidade de Pernambuco, Recife, Brasil.; 4 Secretaria Estadual de Saúde de Pernambuco, Recife, Brasil.; 5 Universidade Federal de Pernambuco, Recife, Brasil.

**Keywords:** Atenção Básica à Saúde, Qualidade da Assistência à Saúde, Unidade Básica de Saúde, Programa Mais Médicos, Avaliação de Programas, Primary Health Care, Quality of Health Care, Health Centers, Health Consortia, Program Evaluation, Atención Básica, Calidad de la Atención de Salud, Centros de Salud, Consorcios de Salud, Evaluación de Programas

## Abstract

O objetivo deste estudo foi caracterizar a qualidade da atenção básica com base nas respostas de profissionais e usuários, focando no tipo de médico - inserido ou não no Programa Mais Médicos (PMM). Realizou-se um estudo transversal aplicando o *Questionário de Fortalecimento da Atenção Básica* a 149 médicos e 795 usuários em unidades básicas de saúde de Pernambuco, Brasil, de fevereiro a novembro de 2018. Neste estudo, foi utilizado o conceito de provimento do PMM no qual foi previsto a destinação do profissional médico para área de vulnerabilidade social. Os médicos foram divididos em três subgrupos: brasileiros não PMM, brasileiros PMM e cubanos PMM. Foram gerados escores médios a partir da perspectiva dos profissionais e dos usuários por meio de análise bivariada e multivariada dos subgrupos dos médicos com variáveis sociodemográficas, educacionais e atributos da atenção primária, considerando-se o nível de significância de 5%. Médicos cubanos do PMM apresentaram os maiores escores médios, especialmente no atributo orientação familiar e comunitária (7,19), seguido pelo subgrupo dos brasileiros do programa (4,74). A análise multivariada mostrou significância para médicos cubanos, sexo feminino e profissionais com residência médica. Entre os usuários, observou-se maior associação com a qualidade da atenção básica no subgrupo dos médicos cubanos, com exceção da longitudinalidade. Os resultados indicam a efetividade dos médicos cubanos do PMM na atenção básica, ressaltando a necessidade de focar na centralidade do usuário. Os achados reforçam a importância de utilizar instrumentos de avaliação precisos e abrangentes na gestão da saúde pública.

## Abstract (en)

This study characterized the quality of primary care based on the responses of professionals and users, focusing on the type of physician - whether or not they were included in the More Doctors Program (PMM, acronym in Portuguese). A cross-sectional study was conducted applying the *Quality and Strengthening of Primary Care Questionnaire* to 149 physicians and 795 users in basic health units in Pernambuco State, Brazil, from February to November 2018. The present study used the concept of PMM provision which foresees the allocation of medical professionals to an areas of social vulnerability. Physicians were divided into three subgroups: non-PMM Brazilians, PMM Brazilians, and PMM Cubans. Mean scores were generated from the perspective of professionals and users through bivariate and multivariate analysis of the physicians’ subgroups with sociodemographic, educational and primary care attributes, considering a significance level of 5%. Cuban physicians in the PMM had the highest average scores, especially in the attribute family and community orientation (7.19), followed by the subgroup PMM Brazilians (4.74). Multivariate analysis showed significance for Cuban physicians, females, and professionals with medical residency. Among users, there was a greater association with the quality of primary care in the subgroup of Cuban physicians, except for longitudinality. Results indicate the effectiveness of PMM Cuban physicians in primary care, emphasizing the need to focus on user centrality. The findings reinforce the importance of using accurate and comprehensive assessment instruments in public health management.

## Resumen

El objetivo del estudio fue caracterizar la calidad de la atención primaria con base en las respuestas de profesionales y usuarios, centrándose en el tipo de médico, incluido o no en el Programa Más Médicos (PMM). Se realizó un estudio transversal aplicando el *Cuestionario de Fortalecimiento de la Atención Primaria* a 149 médicos y 795 usuarios de unidades básicas de salud de Pernambuco, Brasil, de febrero a noviembre del 2018. En el presente estudio, se utilizó el concepto de provisión del PMM, que preveía la asignación de profesionales médicos a zonas de vulnerabilidad social. Los médicos fueron divididos en tres subgrupos: brasileños no PMM, brasileños PMM y cubanos PMM. Se generaron puntajes medios con base en la perspectiva de los profesionales y de los usuarios mediante análisis bivariado y multivariado de los subgrupos de médicos con variables sociodemográficas, educativas y atributos de atención primaria, considerando un nivel de significancia del 5%. Los médicos cubanos del PMM presentaron los puntajes medios más altos, especialmente en el atributo orientación familiar y comunitaria (7,19), seguidos por el subgrupo de brasileños del programa (4,74). El análisis multivariado mostró significancia para los médicos cubanos, mujeres y profesionales con residencia médica. Entre los usuarios, se observó una mayor asociación con la calidad de la atención primaria en el subgrupo de médicos cubanos, con excepción de la longitudinalidad. Los resultados indican la efectividad de los médicos cubanos del PMM en la atención primaria, resaltando la necesidad de centrarse en el usuario. Los hallazgos refuerzan la importancia de utilizar herramientas de evaluación precisas e integrales en la gestión de la salud pública.

## Introdução

A construção histórica da Estratégia Saúde da Família (ESF) visa à reorganização da atenção básica no Brasil, buscando expansão, qualificação e sua consolidação ao garantir melhor acesso e resolutividade na situação de saúde das pessoas e coletividades, além de propiciar um importante impacto na relação custo-efetividade quando segue os preceitos do Sistema Único de Saúde (SUS) ^1^.

Todavia, entre os maiores desafios enfrentados pela organização da atenção básica nos municípios brasileiros, estão a insuficiência e a alta rotatividade de profissionais, especialmente de médicos nas equipes ^2^ ^,^ ^3^. Esses problemas decorrem, entre outras causas, da falta de regulação do Estado sobre a formação e o exercício profissional médico ^4^. Isso porque, ao longo do tempo, o uso corporativo da autonomia da profissão médica atuou em detrimento do papel regulador do poder público ^5^, diferentemente do que acontece na Austrália, Canadá, Espanha e Inglaterra, que estipulam a formação de uma proporção adequada de médicos de família e comunidade ^5^ ^,^ ^6^.

A ausência de políticas públicas de provimento médico tem gerado desassistência à população, principalmente para aqueles que mais precisam de acesso à saúde, configurando um quadro de violência estrutural por inação do Estado ^7^ ^,^ ^8^.

As iniquidades entre as regiões brasileiras, nesse âmbito, já são conhecidas e descritas pelo índice de escassez de profissionais de saúde na atenção básica em relação ao Norte e Nordeste, com, respectivamente, 47,9% e 25,1% dos municípios apresentando escassez ^3^. Por isso, a decisão pública de implantar o Programa Mais Médicos (PMM), com vários eixos de atuação, foi uma resposta necessária de intervenção regulatória do Estado.

O PMM teve como objetivo ampliar e melhorar a atenção à saúde pelos eixos da formação para o SUS e do provimento emergencial de profissionais médicos. O provimento se deu por meio de editais de chamadas nacional e internacional, alocando os médicos nos municípios a partir de critérios que consideravam a situação de extrema pobreza, de escassez ou de ausência de médicos na atenção básica e de vulnerabilidade social nas capitais, cidades do G100, interior e áreas indígenas ^2^ ^,^ ^8^.

Com a lei aprovada pelo Congresso Nacional, o Ministério da Saúde, gestor do PMM, levou cerca de 14 mil médicos para mais de 4 mil municípios brasileiros, entre julho de 2013 e abril de 2014, utilizando médicos brasileiros formados no Brasil e no exterior e médicos estrangeiros ^2^.

Nesse sentido, a cooperação entre Brasil e Cuba no âmbito do PMM representou um marco significativo na história da saúde pública brasileira. Iniciada em 2013, essa colaboração visava endereçar a escassez de profissionais médicos em regiões carentes e remotas do Brasil, onde a dificuldade de acesso à saúde básica era uma realidade. A parceria baseou-se no envio de médicos cubanos para trabalhar nessas áreas, proporcionando não só uma cobertura médica mais ampla, mas também uma oportunidade de intercâmbio cultural e de práticas médicas. Essa iniciativa foi estruturada sob os auspícios de um acordo tripartite entre o Ministério da Saúde do Brasil, a Organização Pan-Americana da Saúde (OPAS) e o governo de Cuba ^9^.

Dessa forma, este estudo visa caracterizar o fortalecimento da atenção básica e analisar sua qualidade por meio do instrumento *Questionário de Fortalecimento da Atenção Básica* (Q-FAB) ^10^, verificando se há diferença entre os tipos de provimento médico do PMM, a partir da ótica dos profissionais e usuários.

## Métodos

Este estudo transversal de abordagem quantitativa aplicou um questionário validado (Q-FAB) nas versões para profissional completa e usuários, no período de fevereiro a novembro de 2018. O estudo foi aprovado, em 2017, pelo Comitê de Ética do Instituto de Medicina Integral Prof. Fernando Figueira (IMIP; CAAE: 698495517.3.0000.5201). Todos os participantes assinaram um Termo de Consentimento Livre e Esclarecido (TCLE). Os profissionais foram informados de que a pesquisa abrangia também os usuários.

Foi calculada uma amostra probabilística do tipo estratificada a partir do porte populacional dos municípios e macrorregiões de saúde no Estado de Pernambuco.

Para tanto, foram utilizadas respostas de 149 profissionais médicos e 795 usuários, que foram selecionados por serem vinculados a unidades de saúde pertencentes aos municípios que foram sorteados a partir do porte populacional. Para participar do estudo, o médico deveria estar há pelo menos seis meses na equipe de saúde da família selecionada e os usuários tinham que ter tido pelo menos duas consultas com o médico selecionado, e ter sido consultado no dia da aplicação do Q-FAB. A decisão de incluir os profissionais e usuários deveu-se à possibilidade realizar triangulação das duas visões sobre o objeto do estudo na perspectiva dos profissionais e dos usuários.

Essa amostra foi estratificada por porte populacional em quatro extratos, nos levando a coletar dados em nove municípios abaixo de 25 mil habitantes, oito municípios entre 25.001 e 50 mil habitantes, três municípios entre 50.001 e 100 mil e três municípios acima de 100 mil habitantes. Sendo sete municípios da macrorregião de saúde I (Região Metropolitana do Recife e Zona da Mata); 11 da macrorregião II (Agreste); três da macrorregião III (Sertão) e dois da macrorregião IV (Vale do São Francisco e Araripe). A seleção dos profissionais e usuários ocorreu de forma pareada nesses municípios, percorrendo unidades que tinham médicos do PMM e unidades sem médicos do PMM e em alguns momentos pesquisamos unidades que tinham médicos dos dois perfis (médicos do PMM e não PMM).

A escolha do Estado de Pernambuco como cenário para nossa pesquisa foi orientada, principalmente, pela localização geográfica do pesquisador principal. Pernambuco, sendo o local de atuação do pesquisador, ofereceu acessibilidade prática e logística facilitada para a realização do estudo. Os municípios e profissionais foram sorteados de forma aleatória, e a seleção de usuários ocorreu de forma pareada em unidades com médicos do PMM, unidades com médicos não PMM e unidades com os dois tipos de provimento.

Na obtenção da amostra, foram utilizados como parâmetros caracterização de maior qualidade e fortalecimento da atenção básica por, pelo menos, 50% dos profissionais, erro alfa de 3%, precisão de 95% e efeito de desenho de 1,2. A partir dos profissionais, derivou-se a amostra dos usuários na proporção de, aproximadamente, 5:1, resultando numa amostra de 149 profissionais e 795 usuários sorteados nas unidades básicas de saúde (UBS).

Para a obtenção da amostra, definimos critérios específicos baseados na qualidade e fortalecimento da atenção básica, como definidos pelo instrumento construído e validado ^10^. Tendo sido estabelecido que pelo menos 50% dos profissionais de cada unidade deveriam classificar sua unidade como possuidora de alta qualidade e fortalecimento na atenção básica. Esses parâmetros foram utilizados para assegurar uma amostra representativa e confiável para o estudo. Para assegurar a representatividade e a precisão da amostra, consideramos um acréscimo de 10% no tamanho da amostra calculada apenas para os médicos, a fim de compensar possíveis perdas amostrais apenas para os profissionais, considerando que a amostra dos usuários foi derivada a partir do atendimento com o médico não tendo ocorrido recusa de participação.

A amostra inicial compreendia 164 médicos e 795 usuários. Após aplicar os critérios de inclusão e exclusão, e considerando as negativas à participação na pesquisa, a amostra final consistiu em 149 médicos e 795 usuários, distribuídos em municípios. Os médicos foram categorizados em três subgrupos: brasileiros não-PMM, brasileiros PMM e cubanos PMM.

Como critério de inclusão, o médico deveria estar há pelo menos seis meses na equipe de saúde da família sorteada e o usuário deveria ter tido o mínimo de duas consultas com o médico selecionado, ter sido consultado no dia da aplicação do Q-FAB e ser maior de 18 anos. O critério de exclusão para os profissionais foi estar afastado por férias ou doença; para os usuários, dificuldade de entendimento do questionário definida como a incapacidade do usuário de compreender as perguntas após duas tentativas de explicação.

O instrumento de pesquisa Q-FAB profissionais versão completa possui 55 itens para resposta. As variáveis com características sociodemográficas somam 10 itens distribuídos em três blocos: (1) características individuais dos profissionais; (2) formação acadêmica; e (3) características do provimento médico.

Os outros blocos somam 45 itens referentes aos atributos da atenção básica ^11^ distribuídos da seguinte forma: um no atributo acesso ao primeiro contato, cinco no atributo longitudinalidade, oito no de integralidade, 20 no de coordenação do cuidado, 10 no de orientação familiar e comunitária e um no atributo de competência cultural. Já a versão Q-FAB usuários contém 33 itens nos seis atributos. Os itens que constituíram esses instrumentos foram compostos por indicadores afirmativos com resposta em múltipla escolha, numa escala Likert de 1 a 5, em que 5 corresponde a maior concordância e 1, a não concordância.

O fortalecimento da atenção primária com base no modelo de avaliação da qualidade de Donabedian e nos atributos de Starfield para a atenção primária à saúde ^11^. Inicialmente, o Q-FAB passou por uma fase de validação de conteúdo, em que especialistas na área de atenção básica avaliaram a pertinência, clareza e relevância de cada item do questionário. Após ajustes baseados em suas recomendações, o instrumento foi submetido a um teste piloto e em uma amostra representativa de unidades de saúde, visando avaliar a aplicabilidade e compreensibilidade dos itens. Posteriormente, a validade de construto do Q-FAB foi estabelecida por meio de análises estatísticas, incluindo análise fatorial exploratória, que confirmou a estrutura dimensional do instrumento. A confiabilidade foi assegurada por meio do cálculo do coeficiente alfa de Cronbach, que demonstrou uma boa consistência interna dos itens em cada dimensão do questionário.

A aplicação impressa das duas versões do Q-FAB foi realizada por profissionais de saúde pós-graduados, bolsistas selecionados e treinados para participar do projeto de pesquisa.

A análise descritiva apresenta tabelas de frequências e das medidas de tendência central e de dispersão. A associação bivariada entre os itens dos atributos em função do provimento médico relacionado ao PMM utilizou o qui-quadrado de tendência (*linear-by-linear association*). Após a checagem da normalidade pelo teste de Kolmogorov-Smirnov da variável dependente de qualidade e fortalecimento da atenção básica, foi realizado o teste de Kruskal-Wallis de associação entre a variável dependente e o provimento médico. A partir do resultado do teste de Kolmogorov-Smirnov foi criada uma variável dicotômica, a qual passou a ser utilizada para alta e baixa caracterização da atenção básica para os valores acima e abaixo da mediana.

Em sequência, realizou-se a análise bivariada entre a variável alta e baixa caracterização da atenção básica e as variáveis sexo, idade, anos de graduado, possuir especialização (Residência Médica) e provimento PMM empregando-se o teste qui-quadrado de Pearson.

Na análise multivariada, foi utilizada a regressão logística para estimativa de *odds ratio* (OR) ajustada e não ajustada, junto a seus respectivos intervalos de 95% de confiança (IC95%), sendo consideradas para o modelo multivariado ajustado todas as variáveis que apresentaram valor de p < 0,05. Para a construção do modelo de regressão logística binária, optamos pelo método ENTER. Nesta abordagem, todas as variáveis consideradas potencialmente relevantes, conforme identificado na análise bivariada, são incluídas simultaneamente no modelo.

O cálculo dos escores para cada um dos atributos ou seus componentes foi realizado pela média aritmética simples dos valores das respostas dos itens do Q-FAB profissionais e usuários após a transformação dos escores da escala Likert em escala numérica de 0 a 10 aplicando a fórmula, na qual o “5” da escala converte-se no valor = 10, e assim sucessivamente: “4” (valor = 7,5), “3” (valor = 5), “2” (valor = 2,5) e “1” (valor = 0) ^12^.

Para avaliar a validação concorrente por meio do grau de concordância entre os escores médios respondidos pelos usuários e os profissionais, em função dos atributos e do padrão de provimento (subgrupos médicos brasileiros não-PMM, médicos brasileiros PMM e médicos cubanos PMM) e suas interações, foram utilizados os seguintes testes estatísticos: teste t de Student, quando anormalidade dos dados foi verificada pelo teste de Shapiro-Wilk, e, quando não houve anormalidade, o teste Mann-Whitney. O teste de Fisher foi empregado para avaliar as interações quando os pressupostos desse teste foram cumpridos, quando não, aplicou-se o teste de Kruskal-Wallis.

A análise e o tratamento dos dados obtidos foram realizados por meio do software SPSS, versão 28.0.1.1 (https://www.ibm.com/) e do software RStudio, versão 4.0.0 (https://rstudio.com/). As informações foram transferidas de maneira anônima para análise.

## Resultados

Importante destacar que, neste recorte específico do estudo, optamos por apresentar exclusivamente os dados provenientes do Q-FAB versão para profissionais de saúde. Essa decisão foi baseada no objetivo de concentrar a análise nos aspectos técnicos e operacionais da prestação de serviços de saúde na atenção básica. No entanto, deve-se registrar que dos 795 usuários que participaram deste estudo, 88,4% eram do gênero feminino, 50,8% estavam na média de 41 anos de idade e 66,3% tinham renda de até 1 salário mínimo.

A Tabela 1 exibe o perfil dos médicos participantes do estudo, segmentados em três grupos: médicos cubanos PMM, médicos brasileiros PMM e médicos brasileiros não-PMM. Entre os médicos cubanos, 69% são do sexo feminino, contrapondo-se aos 52,9% de médicos brasileiros do PMM e 29,9% dos não-PMM. A distribuição etária mostra uma concentração na faixa de 31-40 anos para os médicos cubanos e brasileiros do PMM, enquanto os médicos brasileiros não-PMM são mais jovens, com maior representação na faixa de 20-30 anos.

**Tabela 1:** Perfil dos profissionais entrevistados no estudo. Pernambuco, Brasil, fevereiro a novembro de 2018.

Variáveis	Médicos cubanos PMM		Médicos brasileiros PMM		Médicos brasileiros não-PMM	
	n	%	n	%	n	%
Sexo						
Feminino	33	69,0	18	52,9	20	29,9
Masculino	15	31,0	16	47,1	47	70,1
Idade (anos)						
20-30	16	33,3	10	29,4	16	23,9
31-40	20	41,7	17	50,0	17	25,4
41-50	7	14,6	2	5,9	7	10,4
51-60	5	10,4	2	5,9	6	9,0
61-70	-	-	2	5,9	9	13,4
Acima de 70	-	-	-	-	9	13,4
Não informado	-	-	1	2,9	3	4,5
Estado Civil						
Solteiro/a	18	37,5	14	41,2	20	29,9
Casado/a	25	52,1	19	55,9	40	59,7
Divorciado/a	4	8,3	-	-	5	7,5
Viúvo/a	-	-	-	-	1	1,5
Outro	1	2,1	1	2,9	1	1,5
Nacionalidade						
Brasileiro	-	-	34	100,0	67	100,0
Cubano	48	100,0	-	-	-	-
País de formação						
Brasil	-	-	26	76,5	67	100,0
Cuba	48	100,0	-	-	-	-
Argentina	-	-	1	2,9	-	-
Bolívia	-	-	6	17,6	-	-
Paraguai	-	-	1	2,9	-	-
Tempo de trabalho (em anos)						
Menos de 1	-	-	-	-	2	3,0
1-5	10	20,8	19	55,9	24	35,8
6-10	19	39,6	6	17,6	6	9,0
11-20	12	25,0	2	5,9	5	7,5
Mais de 20	7	14,6	7	20,6	30	44,8
Especialidade						
Médico de família e comunidade	28	58,3	10	29,4	10	14,9
Médico clínico	17	35,4	16	47,1	24	35,8
Outras especialidades	3	6,3	8	23,5	17	25,4
Não possui especialização	-	-	-	-	16	23,9
Município de atuação (macrorregiões)						
Região Metropolitana	21	43,8	13	38,2	30	44,8
Agreste	17	35,4	17	50,0	21	31,3
Serão	6	12,5	4	11,8	11	16,4
Vale do São Francisco	4	8,3	-	-	5	7,5
Município em que reside (macrorregiões)						
Região Metropolitana	20	41,7	13	38,2	26	38,8
Agreste	17	35,4	17	50,0	21	31,3
Sertão	6	12,5	4	11,8	11	16,4
Vale do São Francisco	4	8,3	-	-	5	7,5
Outros estados	1	2,1	-	-	4	6,0
Total	48	100,0	34	100,0	67	100,0

PMM: Programa Mais Médicos.

Fonte: elaboração própria.

Em termos de nacionalidade e formação, todos os médicos cubanos são formados em Cuba, enquanto a maioria dos médicos brasileiros recebeu sua formação no Brasil. Quanto ao tempo de trabalho, há uma variação, com médicos brasileiros não-PMM tendo apresentado experiência acima de 20 anos. A maioria dos médicos atua e reside na Região Metropolitana e no Agreste de Pernambuco (Tabela 1).

Na análise dos atributos em função do provimento médico relacionado ao PMM, 30 itens apresentaram p < 0,05, sobressaindo-se o subgrupo de cubanos PMM como o de maior frequência em 28 dos itens associados. O atributo de coordenação do cuidado teve o maior número de itens, 12. Os brasileiros não PMM tiveram dois itens do atributo Integralidade associados (Quadro 1).

**Quadro 1:** Análise bivariada entre os itens dos atributos em função do provimento médico relacionado ao Programa Mais Médicos (PMM) utilizando o qui-quadrado - profissionais. Pernambuco, Brasil, 2018.

ATRIBUTO	QUESTÃO	VALOR DE P *	CATEGORIA DE MAIOR FREQUÊNCIA
Acesso ao primeiro contato	16 - Frequência de participação no acolhimento	0,004	Cubanos PMM
Longitudinalidade	19 - Participação no mapeamento das condições de vulnerabilidade social da comunidade	0,003	Cubanos PMM
	24 - Verificação de participação em grupos de hipertensos e diabéticos	0,066	Cubanos PMM
	26 - Frequência da participação profissional nos grupos de hipertensos e diabéticos	0,005	Cubanos PMM
	29 - Participação profissional no reconhecimento da importância da UBS para a população	0,467	Cubanos PMM
	37 - Classificação do impacto da solicitação de registros médicos e exames anteriores na prática médica	0,820	Brasileiros PMM
Integralidade	14 - Percentual de tempo destinado a outros procedimentos (que não são consultas médicas)	0,389	Cubanos PMM
	21 - Análise do cartão de menores de 5 anos (vacina e curvas de crescimento)	0,008	Cubanos PMM
	23 - Avaliação de peso e pressão arterial nos adultos	0,002	Cubanos PMM
	27 - Frequência de realização de pré-natal	0,090	Cubanos PMM
	28 - Percentual médio de realização de consulta de puerpério nos últimos 6 meses	0,002	Brasileiros não-PMM
	34 - Percentual de encaminhamentos para consultas de especialistas nos últimos 6 meses	0,053	Cubanos PMM
	35 - Percentual de pacientes que retornam do especialista para acompanhamento na UBS	0,004	Brasileiros não-PMM
	41 - Frequência da consulta/visita ou internamento domiciliar nos últimos 6 meses	0,056	Cubanos PMM
Coordenação do cuidado	20.1 - Participação na discussão de classificação de risco das microáreas nos últimos 6 meses	< 0,01	Cubanos PMM
	20.2 - Participação em reuniões de equipe para discutir os problemas das áreas nos últimos 6 meses	< 0,01	Cubanos PMM
	20.3 - Participação na elaboração do mapa de diagnóstico situacional nos últimos 6 meses	< 0,01	Cubanos PMM
	20.4 - Participação na sala de situação nos últimos 6 meses	0,002	Cubanos PMM
	20.5 - Participação nas discussões com a comunidade sobre problemas de saúde nos últimos 6 meses	< 0,01	Cubanos PMM
	20.6 - Participação no trabalho conjunto com a secretaria para marcação de consultas especializadas nos últimos 6 meses	0,229	Cubanos PMM
	20.7 - Participação no monitoramento de óbitos de menores de 1 ano e morte materna nos últimos 6 meses	0,551	Brasileiros PMM
	20.8 - Participação no recadastramento das microáreas nos últimos 6 meses	0,046	Cubanos PMM
	20.9 - Participação no monitoramento de crianças pré-termo, *near miss* infantil e *near miss* materno nos últimos 6 meses	0,002	Cubanos PMM
	20.10 - Participação nas atividades de busca ativa nos últimos 6 meses	< 0,01	Cubanos PMM
	36 - Frequência da solicitação de registros médicos e exames anteriores nos últimos 6 meses	0,885	Brasileiros PMM
	38 - Frequência de utilização do formulário de referência para encaminhamento ao especialista nos últimos 6 meses	0,696	Cubanos PMM
	39 - Frequência de recebimento do formulário de contrarreferência nos últimos 6 meses	0,970	Brasileiros não-PMM
	44.1 - Frequência com que realizou atividades de articulação com a equipe nos últimos 6 meses	0,001	Cubanos PMM
	44.2 - Frequência com que realizou atividades de articulação com os cuidadores tradicionais nos últimos 6 meses	0,351	Brasileiros PMM
	44.3 - Frequência com que realizou atividades de articulação com os profissionais do NASF nos últimos 6 meses	0,157	Cubanos PMM
	44.4 - Frequência com que realizou atividades de articulação com os profissionais dos demais níveis de atenção nos últimos 6 meses	0,466	Cubanos PMM
	45 - Frequência de realização de diagnóstico epidemiológico do território com a SMS nos últimos 6 meses	0,046	Cubanos PMM
	47 - Frequência de participação em reuniões de planejamento com a ESF para ações de gestão e organização do processo de trabalho da equipe nos últimos 6 meses	0,007	Cubanos PMM
	48 - Frequência de participação de reuniões para autoavaliação nos últimos 6 meses	0,004	Cubanos PMM
Orientação familiar e comunitária	32 - Frequência de convites para participar de atividades locais nos últimos 6 meses	0,002	Cubanos PMM
	42 - Frequência de participação na realização de atividades educativas nas escolas, creches, abrigos ou igrejas nos últimos 6 meses	< 0,01	Cubanos PMM
	43.1 - Participação em atividades educativas externas nos últimos 6 meses sobre planejamento familiar	< 0,01	Cubanos PMM
	43.2 - Participação em atividades educativas externas nos últimos 6 meses sobre prevenção de IST	< 0,01	Cubanos PMM
	43.3 - Participação em atividades educativas externas nos últimos 6 meses sobre saúde bucal	< 0,01	Cubanos PMM
	43.4 - Participação em atividades educativas externas nos últimos 6 meses sobre nutrição	< 0,01	Cubanos PMM
	49.1 - Participação nas reuniões para discussões sobre problemas de saúde da comunidade nos últimos 6 meses	< 0,01	Cubanos PMM
	49.2 - Participação nas reuniões para discussões sobre monitoramento dos indicadores nos últimos 6 meses	< 0,01	Cubanos PMM
	49.3 - Participação nas atividades com o controle social nos últimos 6 meses	< 0,01	Cubanos PMM
	49.4 - Participação nos diálogos com líderes da comunidade para avaliar a satisfação dos usuários nos últimos 6 meses	< 0,01	Cubanos PMM
Competência cultural	51 - Classificação da importância de incorporar conhecimentos acerca de crenças e costumes da comunidade na prática médica pessoal	0,004	Cubanos PMM

ESF: Estratégia Saúde da Família; IST: infecções sexualmente transmissíveis; NASF: Núcleo de Atenção à Saúde da Família; SMS: Secretaria Municipal de Saúde; UBS: unidade básica de saúde.

* Qui-quadrado de tendência (*linear-by-linear association*).

Fonte: elaboração própria.

Na Tabela 2, observa-se que 63,4% do sexo feminino se associou à alta caracterização da atenção básica; em contrapartida, 59% do sexo masculino, à baixa caracterização da atenção básica (p = 0,006). Com relação à especialização, 56,5% dos profissionais com especialização caracterizaram mais altamente a atenção básica, enquanto 83,3% dos médicos sem especialização associaram-se à baixa caracterização da atenção básica (p = 0,002).

**Tabela 2:** Análise bivariada e multivariada entre caracterização da atenção básica e fatores sociodemográficos, educacionais e o tipo de provimento dos profissionais. Pernambuco, Brasil, fevereiro a novembro de 2018.

Variável	Modelo bivariado				Modelo multivariado					
	Caracterização da atenção básica				Não ajustado			Ajustado		
	Baixa (< média) [N (%)]	Alta (> média) [N (%)]	Total [N (%)]	Valor de p *	OR	IC95%	Valor de p *	OR	IC95%	Valor de p *
Sexo				0,006						
Feminino	26 (36,6)	45 (63,4)	71 (47,7)		2,49	1,29-4,82	0,01	2,25	1,03-4,94	0,04
Masculino	46 (59,0)	32 (41,0)	78 (52,3)		1,00			1,00		
Idade				0,222						
< Média	33 (43,4)	43 (56,6)	76 (51,1)							
> Média	39 (53,4)	34 (46,6)	73 (49,0)							
Anos de formação				0,804						
< 7	37 (49,3)	38 (50,7)	75 (50,3)							
> 7	35 (47,3)	39 (52,7)	74 (49,7)							
Especialização				0,002						
Com especialização	57 (43,5)	74 (56,5)	131 (87,9)		6,49	1,79-23,51	< 0,01	5,38	1,34-21,53	0,02
Sem especialização	15 (83,3)	3 (2,0)	18 (12,1)		1,00			1,00		
Provimento				< 0,1						
Brasileiros não-PMM	41 (67,2)	20 (32,8)	61 (40,9)		1,00			1,00		
Brasileiros PMM	20 (50,0)	20 (50,0)	40 (26,8)		2,05	0,90-4,65	0,09	1,47	0,61-3,56	0,39
Cubanos PMM	11 (22,9)	37 (77,1)	48 (32,2)		6,90	2,92-16,29	< 0,01	3,91	1,53-10,00	< 0,01

IC95%: intervalo de 95% de confiança; OR: *odds ratio*; PMM: Programa Mais Médicos.

* Teste qui-quadrado de Pearson.

Fonte: elaboração própria.

Quanto à caracterização da atenção básica, a variável provimento foi estatisticamente significante (p < 0,01): 67,2% dos brasileiros não-PMM se associaram à baixa caracterização da atenção básica, enquanto 77,1% dos cubanos PMM estiveram associados à alta caracterização da atenção básica. As variáveis de anos de formado não demonstraram significância estatística na associação com a caracterização da atenção básica (Tabela 2).

O sexo feminino apresentou mais chance de estar associado à alta caracterização da atenção básica, com OR não ajustado e ajustado de 2,49 e 2,25, respectivamente. O médico com especialização também apresentou maior probabilidade de alta caracterização da atenção básica (OR em torno de 6,49 na versão não ajustada e 5,38 na ajustada).

Ainda na Tabela 2, a análise do provimento médico por subgrupos em relação à alta caracterização da atenção básica identificou que não houve diferença estatisticamente significativa para os brasileiros do PMM e os brasileiros não-PMM nas versões não ajustada e ajustada. Já os cubanos PMM apresentaram associação significativa (p < 0,01) à alta caracterização da atenção básica (OR = 6,90 não ajustada e OR = 3,91 ajustada) em relação aos brasileiros não-PMM.

**Figura 1:**
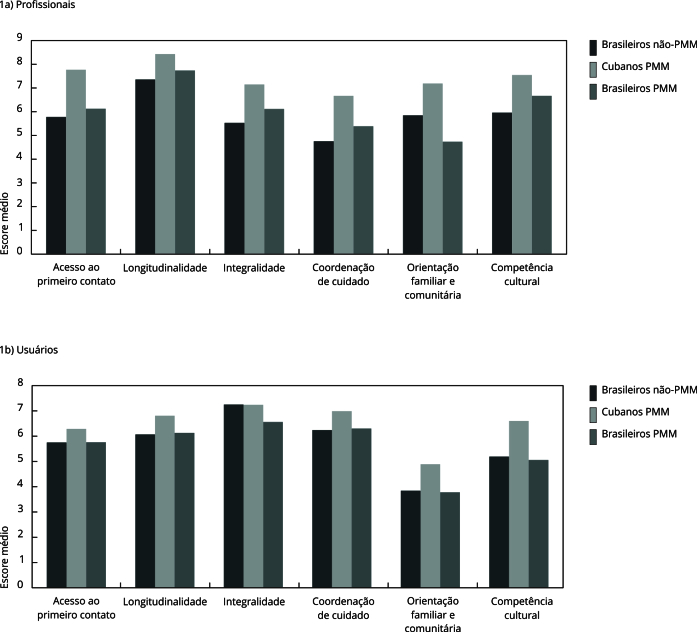
Comparação dos escores médios dos atributos na perspectiva dos profissionais de acordo com o provimento entre as escalas dos profissionais e dos usuários.

Neste estudo, foi identificado que, tanto na perspectiva dos profissionais quanto na dos usuários, o subgrupo cubanos PMM apresentou maiores escores médios em todos os atributos, em comparação aos brasileiros PMM e brasileiros não-PMM, com exceção do atributo integralidade para os usuários (Figura 1).

A análise do grau de concordância e validação concorrente (Tabela 3) apresentou dados estatisticamente significantes no Q-FAB dos profissionais em todos os atributos. Entre os subgrupos de provimento, os maiores escores médios foram verificados no subgrupo médicos cubanos PMM (p < 0,05). O subgrupo cubanos PMM apresentou maior diferença de pontuação em comparação com os demais subgrupos no atributo orientação familiar e comunitária, com escore médio de 7,19, enquanto os não-PMM e os brasileiros PMM apresentaram, respectivamente, 3,85 e 4,74. Na escala dos usuários, no atributo integralidade, os não-PMM (7,25) e os cubanos PMM (7,24) apresentaram escores médios similares. Nos demais atributos, os médicos cubanos PMM tiveram maiores médias.

**Tabela 3:** Grau de concordância e validação concorrente entre as escalas de usuários e profissionais.

Atributos	Brasileiros não-PMM	Cubanos PMM	Brasileiros PMM	Valor de p
	Média (DP)	Média (DP)	Média (DP)	
Acesso ao primeiro contato				
Profissionais	5,78 (2,9)	7,77 (2,1)	6,13 (3,1)	0,0009
Usuários	5,75 (1,6)	6,29 (1,8)	5,76 (1,2)	0,0005
Longitudinalidade				
Profissionais	7,36 (1,1)	8,43 (0,85)	7,74 (1,25)	< 0,001
Usuários	6,07 (2,1)	6,81 (2,1)	6,13 (2,1)	0,0004
Integralidade				
Profissionais	5,53 (1,4)	7,15 (1,1)	6,12 (1,3)	< 0,001
Usuários	7,25 (2,2)	7,24 (2,2)	6,56 (2,1)	0,0025
Coordenação do cuidado				
Profissionais	4,76 (1,5)	6,67 (1,4)	5,39 (1,4)	< 0,001
Usuários	6,24 (3,8)	6,99 (3,9)	6,3 (3,8)	0,0175
Orientação familiar e comunitária				
Profissionais	3,85 (2,1)	7,19 (1,8)	4,74 (1,9)	< 0,001
Usuários	3,84 (3,5)	4,89 (3,5)	3,78 (3,5)	0,0125
Competência cultural				
Profissionais	5,96 (2,9)	7,55 (2,9)	6,67 (3,1)	0,0274
Usuários	5,19 (4,6)	6,6 (4,1)	5,06 (4,5)	0,0015
Média geral - profissionais	5,68 (1,98)	7,44 (1,69)	6,398 (2,01)	
Média geral - usuários	5,71 (2,97)	6,55 (2,93)	5,60 (2,95)	
Valor de p	1,00	0,08	0,12	

DP: desvio padrão; PMM: Programa Mais Médicos.

Fonte: elaboração própria.

Foram comparados os valores médios de escore geral dos atributos entre as escalas Q-FAB dos profissionais e dos usuários por subgrupo de provimento médico, não evidenciando diferenças estatisticamente significativas (Tabela 3).

## Discussão

Em linhas gerais, observamos que os médicos cubanos PMM demonstraram um desempenho significativamente superior em diversos atributos da atenção básica em comparação com seus colegas brasileiros, tanto participantes quanto não participantes do PMM. Além disso, identificamos que, independentemente da nacionalidade, os médicos participantes do PMM tendem a adotar práticas que fortalecem a atenção básica, corroborando a importância do programa na promoção de melhorias nessa esfera do sistema de saúde. Esses resultados são fundamentais para entender as dinâmicas do PMM e seu impacto na atenção básica, fornecendo evidências que podem orientar futuras políticas de saúde e estratégias de fortalecimento da atenção básica no Brasil.

Alguns estudos aplicaram o instrumento PCATool-Brasil (*Instrumento de Avaliação da Atenção Primária*) exclusivamente para profissionais ^13^ ^,^ ^14^ ^,^ ^15^ ^,^ ^16^, ou versão adulto para os usuários ^17^. Portanto, foi inédita a aplicação concomitante, no mesmo estudo, de um instrumento validado para usuários e para profissionais médicos divididos em três subgrupos de acordo com o provimento que avaliasse os atributos da atenção básica.

Outros estudos ^15^ ^,^ ^17^ fizeram análise bivariada isoladamente ou testaram associação, mas nenhum fez a observação item a item.

As análises bivariadas e multivariadas permitiram levantar a associação entre as variáveis dependentes de alta e baixa caracterização da qualidade e fortalecimento da atenção básica e as variáveis exploratórias idade, sexo, tempo de formado, especialização e provimento médico nos três subgrupos, usando as respostas coletadas no questionário aplicado aos profissionais médicos.

Entre os estudos realizados de avaliação da qualidade da atenção básica e o PMM, que aplicaram questionários somente aos profissionais ^13^ ^,^ ^14^ ^,^ ^15^, o estudo de Career et al. ^14^ não encontrou diferença estatisticamente significante entre os resultados de equipes com médicos do PMM e com médicos não-PMM, como se a presença do profissional, por si só, garantisse alto escore para atenção básica. Além disso, atribuíram bons escores à estrutura e ao modelo de atenção das unidades, independentemente da formação do profissional. Todavia, esse estudo contou com 72 profissionais de saúde, entre eles 33 médicos dos quais somente 10 eram médicos do PMM.

Um estudo realizado em nível nacional ^17^ afirma não haver diferença ao medir a qualidade em saúde comparando escores gerais da atenção básica atribuídos por usuários atendidos por médicos brasileiros do PMM, cubanos do PMM e brasileiros não-PMM. Porém, ao analisar mais profundamente os resultados nesse estudo, percebe-se que, no escore de acesso no Brasil e, mesmo no escore geral, na Região Nordeste, houve significância estatística dos valores atribuídos pelos usuários atendidos pelos médicos cubanos do PMM em relação aos brasileiros do PMM e brasileiros não-PMM, reforçando os achados deste estudo.

Corroborando com os nossos achados, estudos com gestores apontam diferenças positivas na atuação dos médicos do PMM, principalmente dos cubanos PMM, tanto para os usuários como para os municípios, garantindo o fortalecimento da atenção básica ^18^ ^,^ ^19^ ^,^ ^20^.

Estudos apontam que a satisfação da população em relação aos médicos PMM apresentou-se elevada, principalmente devido à escuta qualificada das queixas, ao fornecimento de informações necessárias, ao bom atendimento do médico e à existência do profissional, evitando que os usuários se deslocassem para outros municípios em busca de atendimento ^18^ ^,^ ^19^ ^,^ ^20^ ^,^ ^21^. Outro aspecto que contribuiu para a avaliação positiva foi a postura dos médicos: atenciosos, humildes e que demonstravam interesse pela vida do usuário ^19^.

Um estudo conduzido por pesquisadores das cinco regiões do Brasil realizou a análise de diversas bases de dados, bem como pesquisa de campo em 32 municípios de 16 estados brasileiros. Também destacou que o PMM contribuiu, significativamente, para que houvesse médicos em áreas de difícil acesso, colaborando para a garantia de maior equidade no acesso e utilização dos serviços de saúde, uma atenção integral e qualificada, com melhora do acolhimento, vínculo e respeito aos usuários, o que foi determinante para o fortalecimento da atenção básica ^19^.

Estudo desenvolvido por Kemper ^13^ mediu os atributos por meio de escores médios em cada um deles, por escore geral e um escore essencial, utilizando o PCATool-Brasil para profissionais, exclusivamente em 8.235 médicos cubanos da cooperação internacional do PMM. Tanto o escore geral como o essencial indicaram alto grau de orientação à atenção básica. Esse mesmo estudo mostrou que 80,7% dos itens presentes no questionário ficaram acima do ponto de corte de 6,6.

Há de se ressaltar a extensão desse estudo, tendo pesquisado mais de 8 mil médicos cubanos dispostos em 2.713 cidades com situações heterogêneas em termos de estrutura, vulnerabilidade e condições de trabalho. Os escores aferidos entre esses médicos mostraram desempenho semelhante ou superior aos obtidos entre os médicos brasileiros da ESF em outras avaliações fazendo uso do mesmo instrumento, isto é, o PCATool. Esse achado, segundo a autora, pode ser decorrente da formação dos médicos cubanos, todos com especialização em medicina geral e integral, que é como é conhecida a medicina de família e comunidade em Cuba, além do tempo de formação superior a cinco anos e experiência em atenção básica ^13^.

Neste estudo, o subgrupo cubanos PMM teve 77,1% dos seus integrantes com alta caracterização da atenção básica, um índice bastante superior ao dos brasileiros não-PMM, mostrando diferença expressiva no traço latente do Q-FAB.

Com os dados medidos pelo Q-FAB podemos inferir que se sobressaem as médicas, os profissionais que possuem especialização e os oriundos do provimento cubano do PMM. Essa situação aproxima-se do trabalho de Maia et al. ^15^, que encontrou significância estatística ao comparar os escores médios mais altos em todos os atributos obtidos com os médicos cubanos do PMM em relação aos outros médicos não-PMM e enfermeiros.

Maia et al. ^15^ também apresentaram um acréscimo ao achado de Rech et al. ^17^ em relação à especialização, pois profissionais com pós-graduação obtiveram escores médios mais altos do que aqueles sem especialização, embora estatisticamente significante apenas no escore derivado de orientação familiar e comunitária. A pesquisa de Career et al. ^14^ também realizou análise ajustada por idade, sexo, profissão, tipo de instituição formadora, tempo de formação e tipo de provimento profissional, por meio de regressão múltipla que encontrou unicamente médicos cubanos PMM associados de forma positiva aos escores essencial, derivado e geral dos atributos medidos pelo PCATool-Brasil com p < 0,05 nos três escores. Esse achado é comparável ao nosso estudo, embora, na nossa pesquisa, tenhamos aplicado o Q-FAB profissionais somente a médicos.

No Q-FAB profissionais, os cubanos PMM pontuaram com maior diferença no atributo orientação familiar e comunitária, o que evidencia maior integração com as ações da comunidade e, consequentemente, maior visualização dos condicionantes sociais do processo saúde-doença por parte desses médicos.

Apontando, também, nesse caminho, Terra et al. ^22^ observaram, na prática dos médicos cubanos, o conceito da clínica ampliada, refletindo na maior identificação com as condições de vida e de cultura das pessoas da comunidade, as quais são consideradas na elaboração do cuidado de forma horizontalizada.

Ressalta-se a diferença relevante entre os sistemas de saúde, que influencia na atuação dos profissionais: a atenção básica cobre 100% das comunidades e cidades de Cuba, e os médicos residem nas áreas onde exercem sua profissão, o que favorece sua ação transformadora na localidade.

Como importante diferencial do nosso estudo, a análise do grau de concordância e validação concorrente revelou que tanto os profissionais quanto os usuários demonstraram a mesma percepção da caracterização da atenção básica pela escala Q-FAB. Isso afastou o viés de intenção, sendo considerado fator limitante quando um instrumento é aplicado unicamente a profissionais.

À luz das limitações do nosso estudo, percebemos fragilidade na abordagem do atributo acesso ao primeiro contato. Para superar esse ponto, ao fortalecer o modelo conceitual em futuras pesquisas usando o Q-FAB profissionais, será possível agregar perguntas. Quanto ao Q-FAB usuários, os resultados serão analisados e publicados posteriormente. Reconhecemos que, além das limitações já mencionadas, nosso estudo está sujeito a outras potenciais fontes de viés e imprecisão que podem influenciar a validade externa dos resultados. Uma outra limitação é a seleção de amostra restrita a uma única região geográfica, no caso, o Estado de Pernambuco. Embora isso tenha proporcionado uma análise detalhada e contextualizada, limita a generalização dos resultados para outras regiões do Brasil, que podem ter características demográficas, socioeconômicas e de infraestrutura de saúde distintas

Torna-se importante a aplicação do Q-FAB com profissionais em outros estudos, visando agregar mais evidências de validade do seu impacto. Este estudo conclui uma maior caracterização da atenção básica por intermédio do traço latente do Q-FAB pelos médicos cubanos PMM, indicando que, possivelmente, as diferenças entre os médicos avaliados podem ser explicadas pela formação generalista dos médicos cubanos, cujo currículo possui conteúdo equivalente ao do curso brasileiro, no entanto, com foco maior voltado para atuação na atenção básica, além de uma formação internacionalista. Esse fator leva os profissionais a estarem disponíveis para atuação em realidades diversas e, a maioria por ter pós-graduação e experiência de trabalho em outros países, garante uma maior qualidade na oferta do atendimento realizado pelos profissionais cubanos PMM.

## Conclusões

Concluímos que há diferenças significativas na qualidade da atenção básica, conforme evidenciado pelas avaliações do instrumento Q-FAB, especialmente entre os médicos cubanos do PMM e os demais grupos. Essa descoberta enfatiza a efetividade do PMM em melhorar certos aspectos da atenção básica, particularmente por meio da contribuição dos médicos cubanos.
